# Poorly Differentiated Thyroid Carcinoma in a 19-Year-Old Young Female Patient: A Case Report

**DOI:** 10.7759/cureus.65149

**Published:** 2024-07-22

**Authors:** Atul Chavhan, Anjali A Vagga, Prachi Gedekar, Amit Bhoyar, Garima S Agarwal

**Affiliations:** 1 Pathology, Jawaharlal Nehru Medical College, Datta Meghe Institute of Higher Education and Research, Wardha, IND; 2 Biochemistry, Jawaharlal Nehru Medical College, Datta Meghe Institute of Higher Education and Research, Wardha, IND; 3 Medicine, Jawaharlal Nehru Medical College, Datta Meghe Institute of Higher Education and Research, Wardha, IND

**Keywords:** surgery, carcinoma, computed tomography, poorly differentiated thyroid carcinoma, histopathology

## Abstract

Poorly differentiated thyroid carcinoma (PDTC) is a rare type of thyroid carcinoma that develops from follicular epithelial cells. In terms of morphology and prognosis, PDTC falls between well-differentiated thyroid carcinoma (WDTC) and anaplastic thyroid carcinoma (ATC). The spectrum of malignant thyroid tumors originating from follicles ranges from the fatal ATC at one end to the indolent WDTC at the other. We present a case of a 19-year-old female patient complaining of swelling on the right side of the neck. Computed tomography revealed a solid cystic lesion within the right lobe of the thyroid gland. The diagnosis of PDTC was made through histopathological examination. In this case, we evaluated the histological characteristics of the right lobe of the thyroid gland and presented a case report of PDTC in a young female.

## Introduction

The most widely accepted theory of follicular cell carcinogenesis states that thyroid follicular cells can change through multiple stages, resulting in differentiated or undifferentiated thyroid cancer. The progression of follicular-derived thyroid carcinomas from well-differentiated to undifferentiated states has been associated with this approach in numerous phases typified by various molecular changes [1]. Thyroid cancers can be classified based on the initial cells that develop cancer. Anaplastic thyroid carcinoma (ATC) and poorly differentiated thyroid carcinoma (PDTC) are two types of thyroid tumors originating from the endoderm. Anaplastic, follicular, and papillary thyroid cancers are subtypes of differentiated thyroid cancer (DTC), which is a common type of thyroid cancer [2].

PDTC is an extremely rare type of thyroid cancer, accounting for about 2% to 15% of all thyroid cancers. Different approaches to interpreting the histology or the impact of the surrounding environment could lead to differences in the interpretation of thyroid cancer results. Depending on the morphology and clinical features, follicular cell-derived thyroid carcinomas are commonly described as intermediate stages between DTC and ATC [3].

Patients with pathological symptoms indicating the occurrence of thyroid cancer have a five-year survival rate. Even though PDTC and ATC are uncommon, treating patients with these illnesses is essential due to their poor survival rates, highlighting a significant therapeutic need [4]. Aggressive lesions, known as PDTCs, have a severe impact on patients' lives. Proper identification is essential for the next steps in patient care [5].

## Case presentation

We are presenting a case of a 19-year-old female patient admitted to the hospital with a complaint of swelling on the right side of her neck. The patient was stable six months ago when she first noticed a swelling that was insidious in onset and gradually progressive in size. Initially, it was the size of a pea and later progressed to the size of a small lemon. The swelling was not associated with pain, difficulty swallowing, or breathing. All laboratory investigations were conducted, and all lab parameters were normal, as shown in Table 1.

**Table 1:** Lab results and reference ranges for the patient. IPTH, intact parathyroid hormone; FT3, free triiodothyronine; FT4, free thyroxine.

Lab investigation	Patient observed value	Reference value
Hemoglobin %	12.1	12-15 g%
Red blood cell count	4.53	3.8-4.8 million/mm^3^
White blood cell count	9600	4000-11,000 mm^3^
Total platelet count	208,000	150,000-410,000/mm^3^
Serum ionic calcium	4.4	4.65-5.25 mg/dL
FT3	2.70	2.77-5.27 pg/mL
FT4	1.01	0.78-2.19 ng/dL
Thyroid-stimulating hormone	4.7	0.465-4.68 mIU/L
Calcium	8.9	8.4-10.2 mg/dL
IPTH	29.3	7.5-53.5 pg/mL

**Figure 1:**
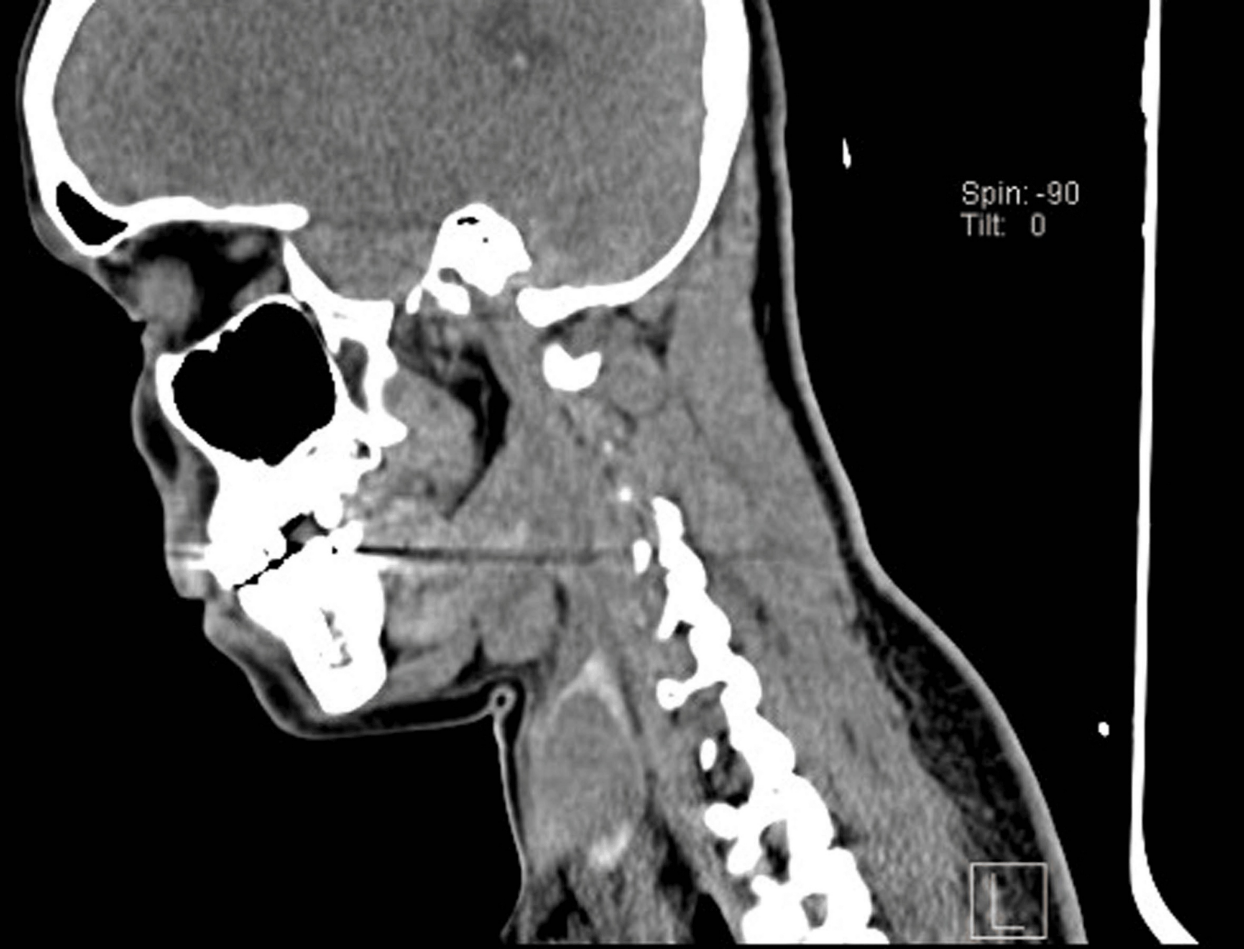
Computed tomography shows a solid cystic lesion within the right lobe of the thyroid.

The computed tomography revealed a solid cystic lesion within the right lobe of the thyroid, as shown in Figure 1.

**Figure 2:**
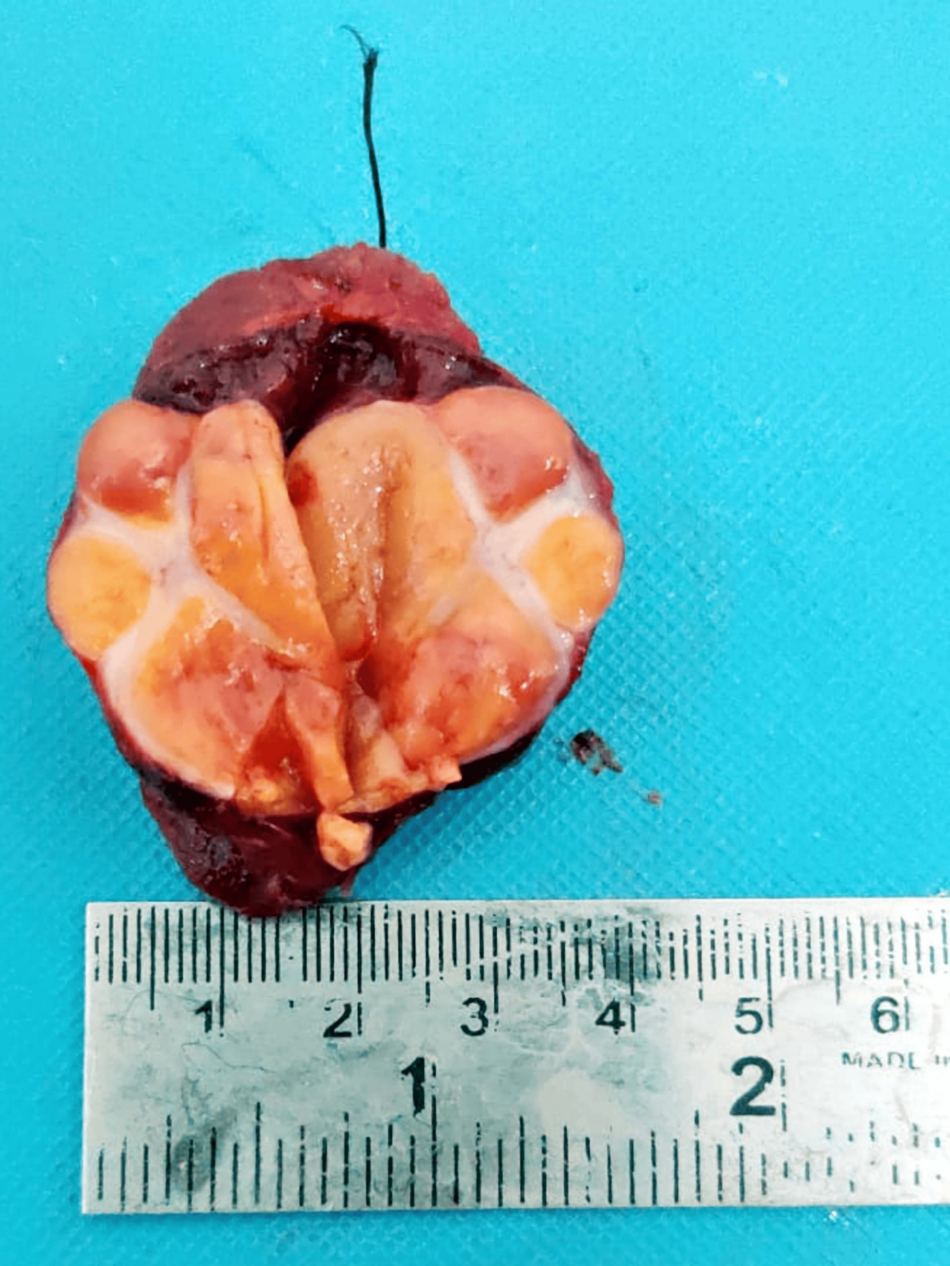
Gross specimen of the right lobe section of the thyroid gland.

A focal capsular breach is noted along the lesion's anterior aspect, suggesting capsular invasion. Anteriorly, it shows a loss of fat planes with the right-sided strap muscle at the site of capsular invasion. Total thyroidectomy was performed. For histopathological examination, the specimen was received in a container labeled as the right lobe of the thyroid gland. On the cut section, the tissue pieces appeared as a grayish-yellow, firm to hard, well-circumscribed growth with septations present, identified as measuring 3 x 2.5 x 0.5 cm, as shown in Figure 2.

**Figure 3:**
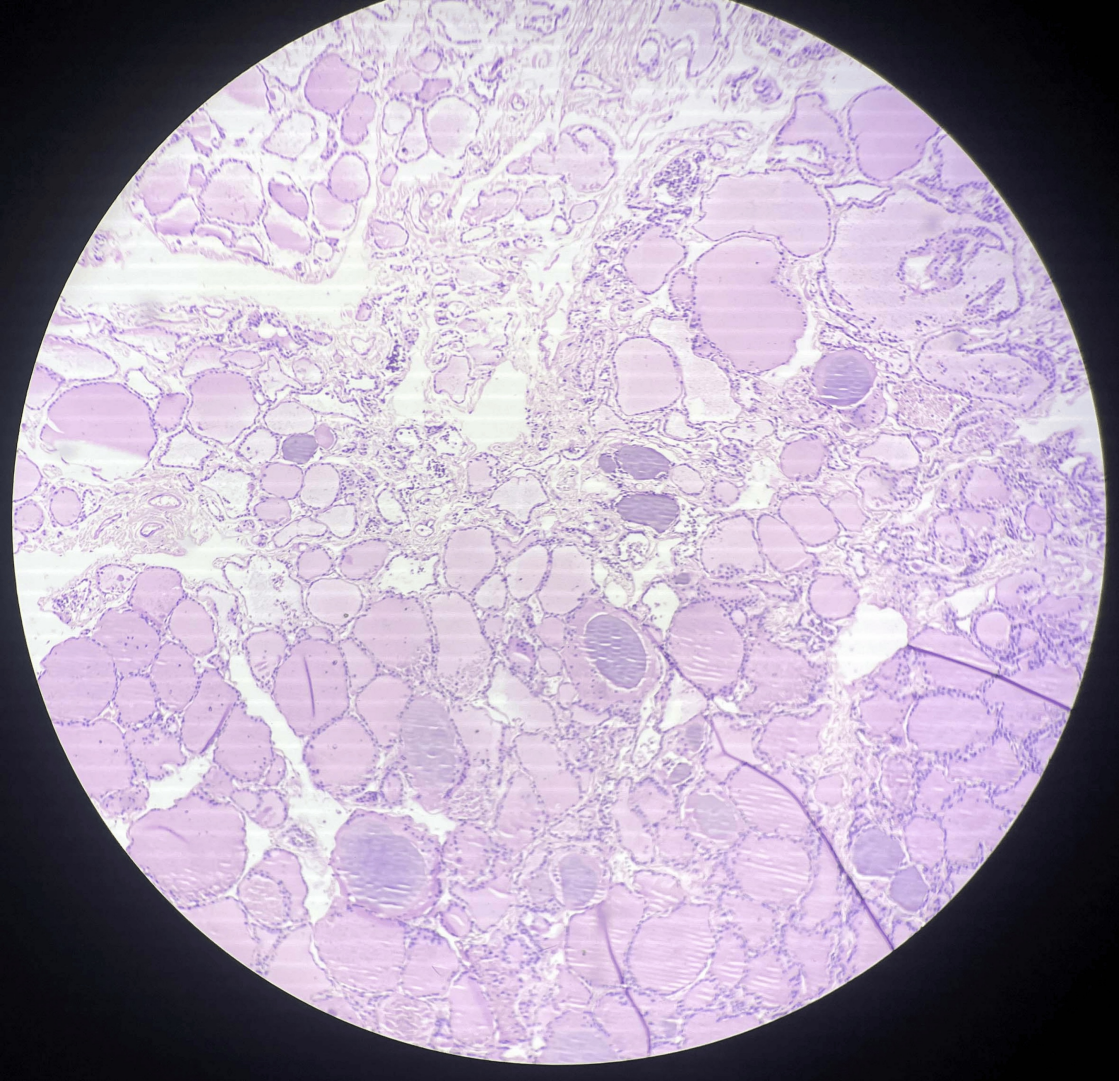
Microscopy shows normal thyroid follicles with colloids.

**Figure 4:**
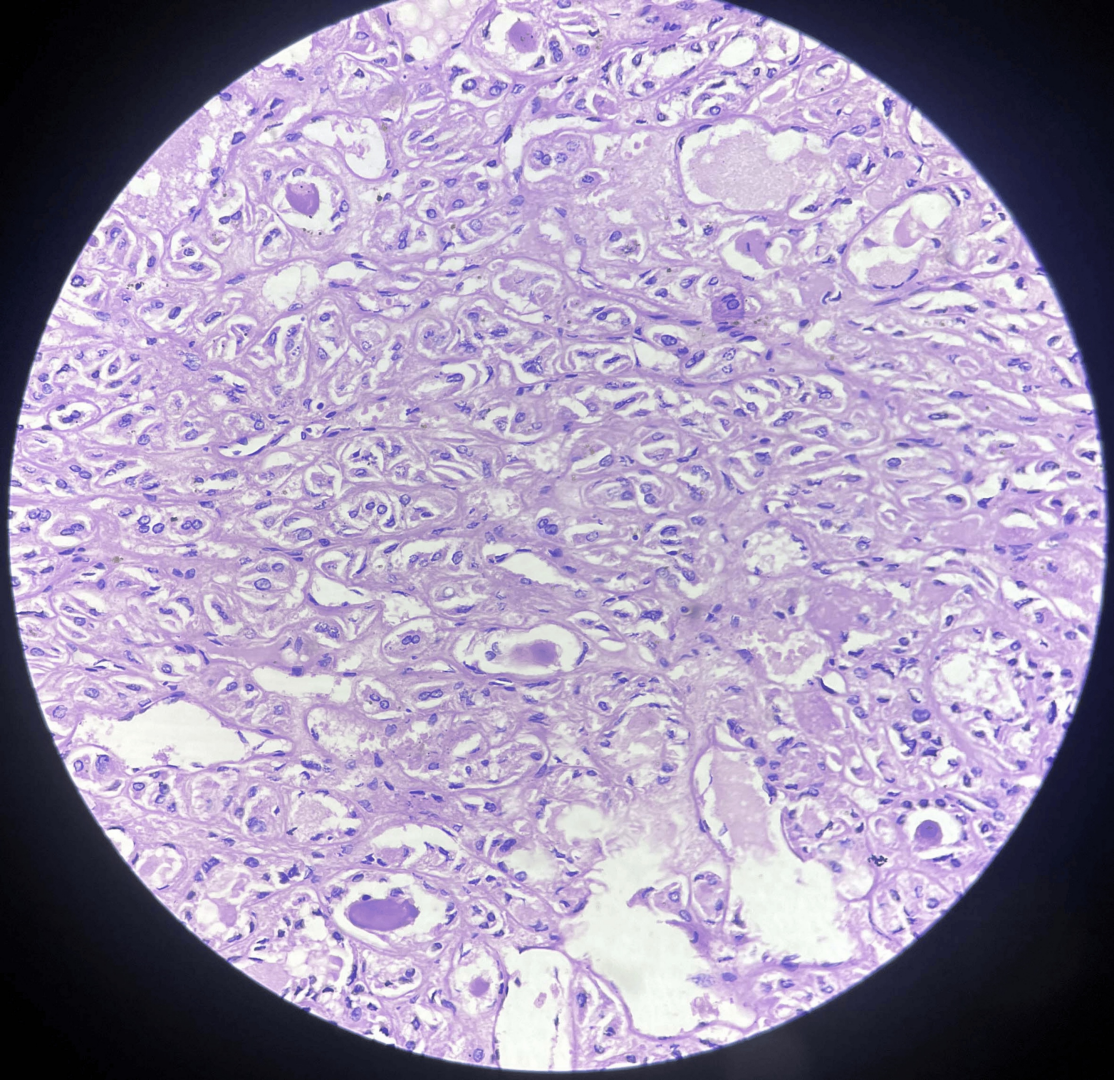
Section shows extensive areas of tumor necrosis with tumor cells arranged in trabeculae separated by vesicular septa. Pleomorphic nuclei are also seen with vesicular chromatin binucleation.

Operative histopathology revealed that the sections from the right lobe of the thyroid show normal thyroid follicles with colloid. The tissue, separated by dense fibrous stroma from a tumor mass, shows extensive areas of tumor necrosis with tumor cells arranged in trabeculae separated by vesicular septa. Pleomorphic nuclei were also seen with vesicular chromatin binucleation noted on histopathology, as shown in Figures 3, 4.

The tumor was classified as American Joint Committee on Cancer (AJCC) stage I. The patient received one dose of IV cephalosporin and levothyroxine. The patient was stable and subsequently discharged.

## Discussion

Thyroid cancer with poor differentiation (PDTC) is still a contentious diagnosis. Some practitioners refer to thyroid cancers that do not trap radioiodine on radioiodine scans (either at the beginning or throughout monitoring) as PDTC [6]. In contrast, others think it more suitable to refer to fast-developing thyroid tumors that have not responded to radioactive iodine therapy. However, assessing whether radioiodine therapy has succeeded is sometimes challenging [6]. According to a model of carcinogenesis established by Landa et al., well-differentiated tumors give rise to PDTCs and ATCs through the accumulation of significant additional genetic anomalies, many of which have prognostic and potentially therapeutic significance. When ATC and PDTC are compared, the extensive genomic alterations in ATC highlight their increased pathogenicity and mortality [7].

Ibrahimpasic et al. conducted a study suggesting that PDTC patients could benefit from adequate surgery, adjuvant therapy, and great locoregional control. Disease-specific deaths in this series are usually caused by uncontrollably occurring locoregional recurrence rather than by distant metastases [8]. Bellini et al. discovered that due to the difficulty in detecting such types of cancers, poorly differentiated thyroid cancer may go undiagnosed. Determining the percentage of poorly differentiated illness is essential for precise estimation of recurrence prevention and medication planning. The Turin proposal, which comprises an insular or trabecular pattern with a high mitotic index, is the most commonly used diagnostic technique [9]. According to Colombo et al., lobectomy can be recommended for low-risk microcarcinomas, and in a small percentage of cases, further treatments are necessary. Therefore, in contrast to individuals treated with a complete thyroidectomy, a longer follow-up time is usually required to demonstrate an event-free result. However, despite the higher probability of postoperative complications, total thyroidectomy should be preferred for intermediate-risk micro- and macro-DTCs in order to obtain a favorable response [10]. Genetic counseling plays a crucial role in thyroid carcinoma by examining family history related to risk assessment and management.

## Conclusions

This case study highlights the occurrence of PDTC in a 19-year-old female. A CT scan revealed a solid cystic lesion within the right lobe of the thyroid. Histopathology showed tumor necrosis with tumor cells arranged in trabeculae separated by vesicular septa. PDTC is recognized as an uncommon condition with a poor prognosis and challenging treatment. Therefore, for proper care and a better prognosis, it is crucial to identify and differentiate this histological variety. This case underscores that obtaining locoregional control in PDTC with the proper surgical procedure and adjuvant treatment is possible. Practical analysis based on pathological diagnosis and radiology is essential for therapeutic decision-making and post-treatment monitoring. Further research and discussion into the diagnosis and management of the disease, as well as proper medication, offer hope for improving outcomes in patients with PDTC.
